# High—throughput and automated screening for COVID-19

**DOI:** 10.3389/fmedt.2022.969203

**Published:** 2022-09-15

**Authors:** Nestor Jonguitud-Borrego, Koray Malcı, Mihir Anand, Erikan Baluku, Calum Webb, Lungang Liang, Carlos Barba-Ostria, Linda P. Guaman, Liu Hui, Leonardo Rios-Solis

**Affiliations:** ^1^ Institute for Bioengineering, School of Engineering, The University of Edinburgh, Edinburgh, United Kingdom; ^2^ Centre for Synthetic and Systems Biology (SynthSys), The University of Edinburgh, Edinburgh, United Kingdom; ^3^ School of Biochemical Engineering, Indian Institute of Technology BHU, Varanasi, India; ^4^ School of Bio-Security, Biotechnical and Laboratory Sciences Makerere University, Kampala, Uganda; ^5^ BGI Clinical Laboratories, BGI-Shenzhen, Shenzhen, China; ^6^ Escuela de Medicina, Colegio de Ciencias de la Salud Quito, Universidad San Francisco de Quito USFQ, Quito, Ecuador; ^7^ Centro de Investigación Biomédica (CENBIO), Facultad de Ciencias de la Salud Eugenio Espejo, Universidad UTE, Quito, Ecuador; ^8^ School of Natural and Environmental Sciences, Newcastle University, Newcastle upon Tyne, United Kingdom

**Keywords:** diagnostic, COVID-19, high-throughput, automation, SARS-coV-2

## Abstract

The COVID-19 pandemic has become a global challenge for the healthcare systems of many countries with 6 million people having lost their lives and 530 million more having tested positive for the virus. Robust testing and a comprehensive track and trace process for positive patients are essential for effective pandemic control, leading to high demand for diagnostic testing. In order to comply with demand and increase testing capacity worldwide, automated workflows have come into prominence as they enable high-throughput screening, faster processing, exclusion of human error, repeatability, reproducibility and diagnostic precision. The gold standard for COVID-19 testing so far has been RT-qPCR, however, different SARS-CoV-2 testing methods have been developed to be combined with high throughput testing to improve diagnosis. Case studies in China, Spain and the United Kingdom have been reviewed and automation has been proven to be promising for mass testing. Free and Open Source scientific and medical Hardware (FOSH) plays a vital role in this matter but there are some challenges to be overcome before automation can be fully implemented. This review discusses the importance of automated high-throughput testing, the different equipment available, the bottlenecks of its implementation and key selected case studies that due to their high effectiveness are already in use in hospitals and research centres.

## Introduction

The COVID-19 pandemic has become an enormous challenge for the health systems of many countries. As of today, according to information provided by the World Health Organization (WHO, https://covid19.who.int, 08/05/2022) it is calculated that over 6 million people have lost their lives and 530 million more have tested positive for the virus. Studies have suggested that approximately 15.6% of people infected are asymptomatic and may not be aware they carry the virus (1), making them potentially deadly vectors of infection.

The European Centre for Disease Prevention and Control, https://www.ecdc.europa.eu/en/publications-data/contact-tracing-covid-19-evidence-scale-up-assessment-resources, April 2021) advises that an effective pandemic control strategy requires robust testing as well as a comprehensive track and trace process for positive patients. Despite these recommendations, the high infection rate of SARS-CoV-2 has led to rampant spread of the disease and consequently a huge demand for diagnostic testing. To meet this demand and increase the testing capacity of communities worldwide, automated workflows have come into prominence as they enable high-throughput screening, faster processing, exclusion of human error, repeatability, reproducibility and diagnostic precision (2).

This review discusses the importance of automated high-throughput testing, the different equipment available, the bottlenecks of its implementation and key selected case studies that due to their high effectiveness are already in use in hospitals and research centres. We also cover the development and automation of different protocols for SARS-CoV-2 testing, highlighting their advantages and disadvantages as well as considering their impact in the COVID-19 pandemic.

Even though vaccination campaigns across the world have been initiated, it will take a long time before most people are vaccinated against SARS-CoV-2. Additionally, manual testing has limited capacity in terms of number of tests processed, while for high-throughput testing it can reach a few thousand tests per day depending on the setup (3). While vaccine and testing campaigns are ongoing, many companies at the cutting-edge of computer-aided biology like Analytik Jena (Germany), Beckman Coulter (USA), Hamilton (USA) and Tecan (Switzerland) have been donating their time and effort to provide automated testing solutions for SARS-CoV-2 diagnosis, as the crisis is still at a peak in many countries (Computed aided biology, https://www.computeraidedbiology.com/cab-companies-on-covid19, 03/21/2021).

In addition to this, to maximise test processing, high-throughput testing is usually combined with other molecular diagnosis developments that could potentially help to reduce not only testing times but also hand labour and potentially the need for costly, additional equipment. For instance, although RT-qPCR is considered by academia to be the “gold standard” for SARS-CoV-2 testing (4), novel techniques have been developed or adapted for COVID-19 testing based on the latest developments in emerging fields such as synthetic biology. Some examples of these alternative point-of-care methods include workflows based on the reverse transcription loop-mediated isothermal amplification assays (RT-LAMP) (5, 6) and CRISPR-based tests (7) which could potentially drastically reduce the consumables required and capital costs while maintaining the same testing efficiency.

## SARS-CoV-2 testing methods

### RT-qPCR

Until now, the RT-PCR real time reverse transcription- polymerase chain reaction has been the gold standard for COVID-19 diagnosis worldwide (8). It is used to amplify a sequence of DNA using the polymerase chain reaction (9). In the diagnosis of SARS-Cov-2, target genes are usually *E* gene (oligonucleotide sequence of envelop gene) and *N gene* (nucleocapsid gene). The RNA-dependent RNA- Polymerase gene (RdRp/Hel) is also targeted to confirm the presence of COVID-19 in the patient sample (10) and in some other labs *S* gene and Orf1ab have also been used (11). Due to RT-qPCR's expensive instrumentation requirements and time-consumption, alternatives methods have been also developed for rapid detection.

### RT-LAMP based diagnostics

The Reverse transcription loop-mediated isothermal amplification RT-LAMP based diagnostic was initially developed for the Middle East Respiratory Syndrome coronavirus (MERS-Cov) (12). It is used for viral genetic pathogen diagnostics (mainly RNA viruses) as it takes an hour to complete the amplification process, considerably less than the RT-PCR method. In RT-LAMP, there are strands replacing RNA polymerase and primers which amplify the specific DNA sequence of the virus. This technique has been adapted for diagnostics of SARS-CoV-2 with a limit of detection (LOD) 100 RNA copies/ reaction (13).

## CRISPR based diagnostics for COVID-19

CRISPR-based diagnostics have been introduced as a feasible method of high-throughput testing. CRISPR techniques are highly adaptable and vary depending on the situation (3). To automate these systems, it is important to understand some of the key steps needed to be performed in such diagnostic systems:

• Isothermal amplification method: recombinase polymerase amplification (RPA or reverse-transcription loop mediated isothermal amplification (RT-LAMP)
• CRISPR enzyme used: Cas9 and Cas12 for DNA detection, Cas13a for RNA detection.
• Additional steps for *in vitro* transcription if RNA is detected sensed
• Results visualisation method (gel electrophoresis, fluorescence, lateral flow strips, naked eye)

Following these different options, different CRISPR based SARS-CoV-2 detection methods have been developed with great potential for automation. The next section discusses four of the most promising solutions.

### SHERLOCK STOPCovid

**Figure 1:**
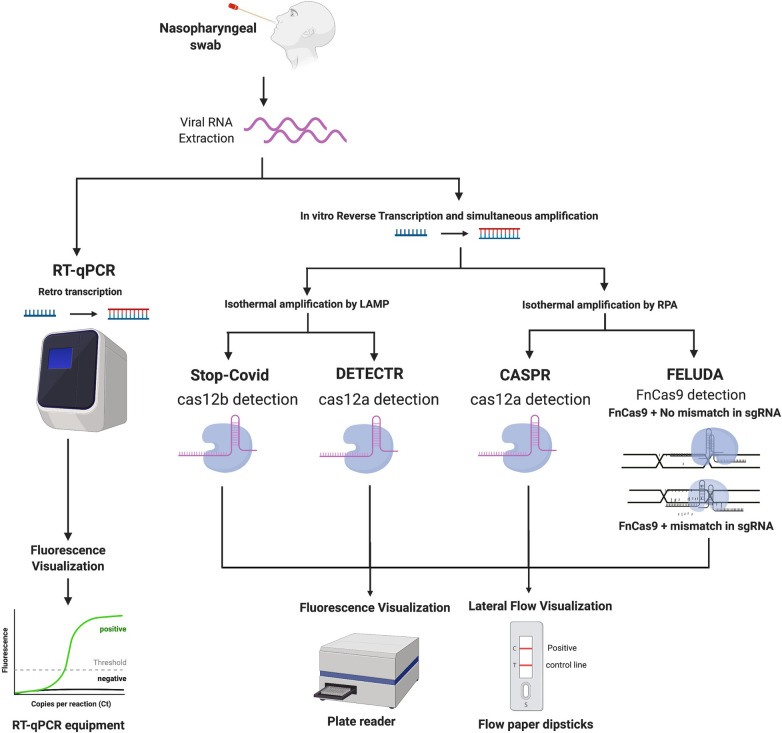
(created with BioRender.com). Workflow comparing different diagnostic methodologies for COVID-19. Images describe a general overview of how each method works, starting from the sample taking and then to viral RNA extraction. From this point, the next step is either direct amplification or retro transcription into DNA for further detection using different methods, potentially RT-PCR or CRISPR-based analysis, followed by the visualisation step.

The Specific High Sensitivity-Enzymatic Reporter UnLOCKing (SHERLOCK) was originally developed by the Fang Zheng laboratory in order to detect cases from both the Dengue and Zika Virus (14). SHERLOCK STOPCovid is an adaptation of the original method for COVID-19 detection (7) and it includes three key steps (Figure 1):

1) Lysis of virus contained in patients sample using QuickExtract for viral RNA extraction.
2) Detection of the virus using the STOPCovid reaction. During this step RT-LAMP is combined with Cas12b for viral detection (instead of the Cas13a originally used in SHERLOCK).
3) Results visualisation using lateral flow paper dipsticks which captures the cleaved reporter RNA with labelled ends on specific antibody bands. For high-throughput processing the readout step can also be performed by fluorescence, using a DNA reporter.

The LOD of STOPCovid is 100 copies of SARS-CoV-2 RNA per reaction (7).

### DETECTR

DNA Endonuclease Targeted CRISPR Trans Reporter (DETECTR) method was reported by Broughton et al. (15) and can be divided into the following steps (Figure 1):

1) Viral RNA amplification by RT-LAMP.
2) Cas12a identification of the SARS-Cov-2 sequence, and further reporter molecule is cleaved, indicating presence of SARS-CoV-2 in the sample.
3) Results visualisation using lateral flow paper dipsticks or plate reader.

The LOD for this diagnostic method is 10 copies/µl reaction.

A similar approach was used by Malcı et al. (16). In this study a One-Pot COVID-19 CRISPR/Cas12a-RPA reaction was performed and optimised using design of experiments (DoE).

Results revealed that addition of reverse transcription buffer and RNase inhibitor (compounds usually omitted in one-pot reactions) can significantly improve the performance of the reaction (16). Very importantly, the authors suggested that the process is highly scalable using automation and high-throughput testing.

### CASPR Biotech's

The CASPR Biotech's (Argentina) method (17) involves the following steps (Figure 1):

1) Viral RNA amplification by RPA technology.
2) Targeted DNA detection using Cas12a.
3) Results visualisation using lateral flow paper dipsticks or plate reader.

The LOD of this assay is 10 copies/µl in both fluorescence and lateral flow dipstick (17).

### FELUDA

The FnCas9 Editor Linked Uniform Detection Assay (FELUDA) is a CRISPR Cas9 based method developed by Azhar et al. (18). The steps used in this method involve (Figure 1):

1. RNA extraction
2. Viral RNA amplification by PCR using biotinylated primer, which is immobilised on beads containing streptavidin coating. Amplification can also be performed by RT-RPA amplification method.
3. Fluorescence-labelled Cas9 complexes have sgRNA that interacts with immobilised target sequence.
4. Analytical signals generated and visualised using a streptavidin coated lateral flow dipstick.

FELUDA reached a LOD of ∼10 copies of purified viral sequence after optimizing PCR conditions. When coupled with RPA LOD is ∼400 copies of starting RNA substrate per *µ*l (18).

To end this section, it is important to highlight some of the advantages and disadvantages that these methods have in comparison to the standardized and widely used RT- PCR and RT-LAMP. All the CRISPR technologies mentioned above have a huge potential to be automated at a lower cost which may be an important advantage compared to traditional methods in lower income communities for high-throughput diagnostics. This is due that many of these diagnostic technologies can work at a single temperature with minimal equipment requirements and complexity (7). However, one of the biggest disadvantages of these new diagnostic methods is the availability and cost of some of reagents (16), whereas reagents for RT-PCR and RT-LAMP can be purchased from most of the retailers in the field at bulk cost. We anticipate that current efforts in lowering the cost of such reagents will provide some relieve to the shortage of reagents in the near future for these types of technologies (19).

Sensitivity is also an important aspect, according to literature, some of these CRISPR based methods can offer LOD as low as 10 copies/µl (15, 17) which is still one order of magnitude higher than the traditionally used techniques of RT-PCR with a LOD of ∼0.1 copies of viral RNA per µl of transport media (20) and RT-LAMP with LOD of ∼6.5 RNA copies/µl (13). Future work optimising the CRISPR assays such as the one performed by Malcı et al. (16) could further reduce the LOD to match similar levels as the traditional methods.

Finally, it is important to remark that despite that the previous methodologies have followed the sampling and testing approvals and procedures in accordance with recommendations from regulations agencies such as the FDA, CDC (7) and the WHO (15), clinical testing have only been done for research purposes as the approval for commercial purposes would further require validation and approval from the corresponding sanitary authorities. An exception to this was made for the SHERLOCK protocol as it was granted Emergency Use Authorization by the FDA tom carry out testing. However, this was only limited to laboratories certified under the Clinical Laboratory Improvement Amendments of 1988, that meet requirements to perform high complexity tests (FDA, https://www.fda.gov/media/137746/download, 23/08/2022).

## Automation at the epicentre of the outbreak

**Figure 2:**
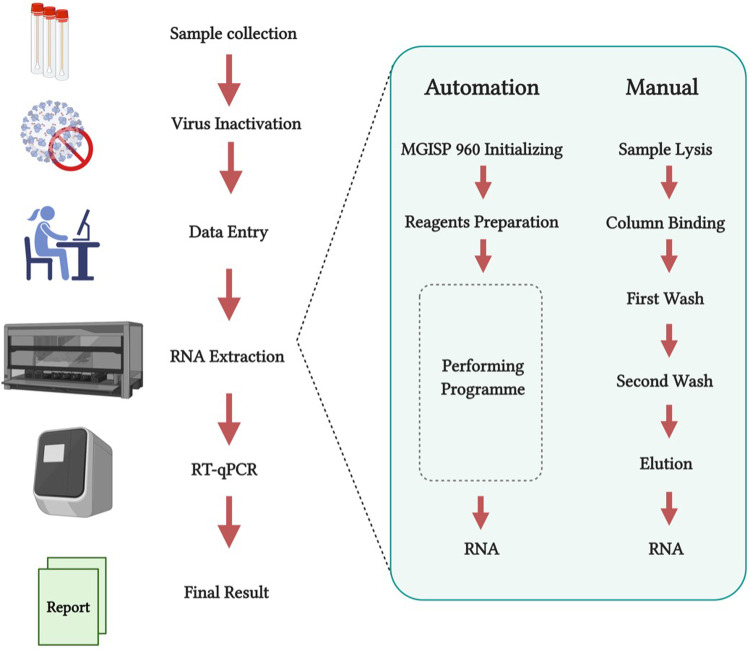
(created with BioRender.com). RT-qPCR test workflow comparing both manual and automated nucleic-acid extraction in Huo-Yan Lab. Automation platforms increase testing capacity whilst simultaneously decreasing processing time. Modified from Liu et al. (22).

One of the most prominent examples of automation support for SARS-CoV-2 testing and screening was at the origin of the outbreak in Wuhan, China. By the 9th of February 2020, Huo-Yan Lab (or Fire Eye Lab) managed to perform 14,000 tests per day by completing an automated extraction of nucleic-acid as of part of the RT-qPCR testing workflow. By the 1st of March 2020, less than a month later, capacity was increased to 20,000 tests per day (21). In this workflow (Figure 2), the authors used the MGISP-960 automated platforms (MGI, China). RNA extraction was performed using MGI's (China) MGIEasy Magnetic Beads Virus DNA/RNA Extraction Kit to get high-throughput and standardised clinical testing as shown in Figure 2 (22). To compare the efficiency of the procedure, manual extractions were completed by using the QIAamp Viral RNA Mini Kit. Manual processing took 1 h and 50 min for 24 samples while comparatively just 1 h and 8 min was required to process 192 samples using automated extraction. Another impressive platform developed by BGI (China) is the MGISP-NE384. This platform is another high-throughput automated nucleic acid extractor which has adopted magnetic rod technology, allowing processing of 384 samples in only 35 min (BGI Genomics, China). These results indicate that automation is key for a successful strategy against SARS-CoV-2.

## Automation of alternative SARS-CoV-2 testing methodologies

The alternative testing techniques described in Figure 1 have shown promising results due to their simplicity, good limit of detection and the lack of need for expensive equipment (7, 15, 18). These reasons make them perfect candidates to be used in high-throughput testing. As a response to the ever-growing needs for rapid COVID-19 testing, many biofoundries which can be described as automation facilities with the capacity to design, test and build biological constructs on different scales, have offered their input on how to use their automation platforms for mass, high-throughput SARS-CoV-2 testing.

**Figure 3:**
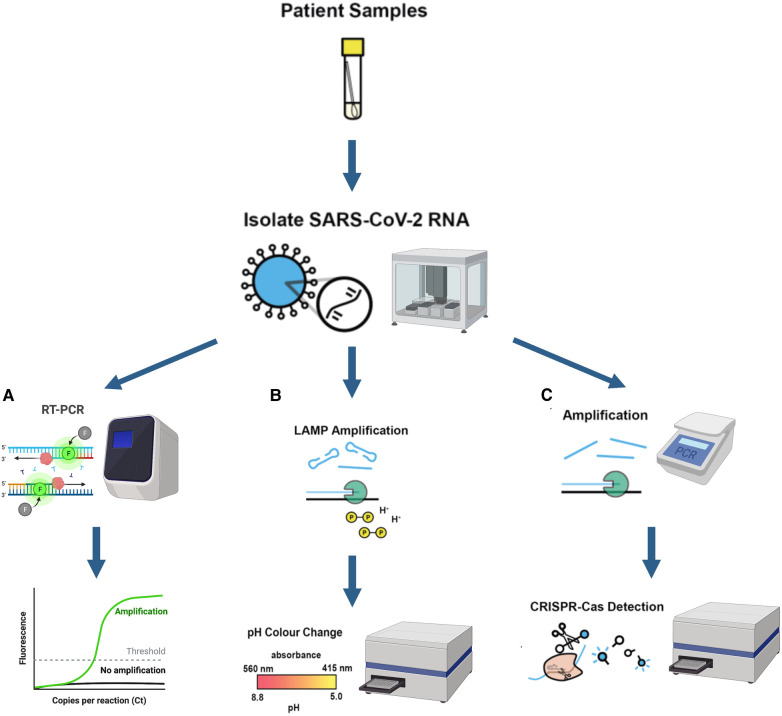
(created with BioRender.com). Schematic illustration of three different automated diagnostic workflows from patient samples and their corresponding required equipment (**A**) RT-PCR diagnostic workflow (**B**) LAMP diagnostic workflow (**C**) CRISPR-Cas13a nucleic acid detection workflow Equipment used for each workflow is also shown in the figure. A qPCR device is a necessity for RT-qPCR work while the plate reader is used for CRISPR-Cas and LAMP workflows to detect absorbance change. Figures modified and adapted from Crone et al. (2).

A perfect example of this is the work carried out at the London Biofoundry, where three different automated workflows were developed. By using Analytik Jena's (Germany) CyBio FeliX and Labcyte's (USA) Echo 525 liquid handling platforms, workflows involving RT-qPCR, CRISPR-Cas13a, and LAMP were automated for SARS-CoV-2 detection (2). As previously mentioned, both Cas13a and LAMP methods were shown to be innovative alternatives that eliminated the need of a qPCR device, reducing both equipment and reagent cost. Another advantage was the reduction in complexity, making it simpler to perform and analyse the results for non-specialised personnel, as well as easier to automate. Diagrams of these two novel detection approaches are shown in Figure 3.

The automated process was divided into two stages for each diagnostic methodology: RNA extraction and then amplification steps. The first step was carried out by CyBio FeliX machine (Analytik Jena, Germany) while the latter was performed by Beckman's (Beckman Coulter Company, USA) Echo 525. Two different RNA extraction kits from Analytik Jena and Promega and three different qPCR master mixes from ThermoFisher (TaqPath and Fast Virus) and NEB (Luna) were also tested.

The authors prepared dilutions of virus-like particles (VLP) containing 5, 25, and 250 copies per reaction to test for the sensitivity of the automated methodologies. This allowed the development of the automated workflow to be sped up without needing to have a constant supply of clinical COVID-19 patient samples, which would require Biosafety Level 2 Laboratory (BSL-2) in order to handle them. Using the VLP also allowed comparing the methods using well controlled and characterised samples for the three different diagnostic methods. It was observed that the detection threshold when using LAMP was at least 30 copies of VLP, while for CRISPR and qPCR the detection threshold was 2.5 VLP.

The automated RT-qPCR workflow was also validated with 173 patient samples obtained from Northwest London Pathology (NWLP). A comparison between the qPCR workflow developed with the selected RNA extraction kit (Analytik Jena innuPREP Virus DNA/RNA Kit) to that used at that time by the NWLP (a multiplexed- tandem PCR workflow) was made. A good correlation (*R*^2^ = 0.8310) was shown between the results given by these two workflows. A second validation was made to further expand the workflow for its use with RNA extraction kits from different suppliers. On this occasion the Analytik Jena innuPREP Virus DNA/RNA Kit previously validated was compared with the Promega Maxwell HT Viral TNA extraction kit. A high correlation (*R*^2^ = 0.9357) was obtained between the results given by these kits. Finally, the automated RT-qPCR described above, which was the only FDA-validated method at the time of its development, was put into operation in two London hospitals with a capacity of 2,000 tests per day.

### Microfluidic COVID Testing

Lab-on-a-chip technologies and microfluidic systems have been increasingly used in various applications within biotechnology as they offer unique advantages such as portability, precise liquid control and low reagent requirement (23). Therefore, microfluidic technology can accelerate conventional biochemistry-based tests especially for high-throughput testing with lower sample volumes. In the last decade, many automated microfluidic molecular diagnosis platforms have been developed and some of them are also commercially available for field use (24).

Microfluidic systems have been also adapted to develop alternative SARS-CoV-2 detection methods. Ramachandran et al. used on-chip electric field control for automated nucleic acid purification in their CRISPR/Cas12a mediated COVID-19 detection method (25). Following an off-chip RT-LAMP isothermal amplification, the selective ionic focusing technique was implemented on a microfluidic chip to purify the nucleic acid templates to be targeted by Cas12a/gRNA complexes to produce fluorescence signals. Researchers reported more than 96% accuracy on 64 clinical samples using this integrated microfluidic system (25). RT-LAMP was also used for automated nucleic acid amplification in a centrifugal microfluidic system (26). After sample preparation, this platform performed a fully automated process from sample-in to answer-out using centrifugal force for nucleic acid separation in a microfluidic disc (26). For the detection of multiple respiratory tract pathogens including SARS-CoV-2, a microfluidic chip-based PCR-array system, Onestart, was developed using magnetic force for nucleic acid purification (27). Onestart was able to complete the sample-in-answer-out process including lysis of samples, nucleic acid extraction and amplification and result output in a fully automated manner. The study reported consistent results with real-time PCR with 100% specificity in 21 different pathogens (27).

Apart from nucleic acid-based detection methods, microfluidic systems have been also employed for serology assays. A computer-controllable semi-automatic microfluidic device has been developed for SARS-CoV-2 antigen detection (28). The device consisted of 200 microchambers for high-throughput testing and was capable of detecting the whole spike antigen with 95% sensitivity in clinical samples (28). Moreover, an automated microfluidic platform has been developed for anti-SARS-CoV-2 antibody testing (29) as COVID-19 antibody tests can be used to obtain important information about the patient's medical history (30). The platform, named automated ELISA on-chip, was used to detect antibody levels of COVID-19 patients and vaccinated individuals. The photos taken by smartphones were analysed by an image processing software and comparable results with the traditional ELISA on microplate method were obtained (29).

Automated microfluidic technologies have a great potential to increase the accessibility of COVID-19 diagnostic tests and to accelerate high-throughput detection processes, especially for POC testing. In addition, the automated microfluidic platforms developed for other pathogens (31), can be readily adapted to be used for COVID-19 diagnosis. In this way, more alternative methods might be available to be used for the POC testing where the access to the sophisticated instruments is limited.

## Free and open source scientific and medical hardware (FOSH)

With the onset of the outbreak worldwide, many biotech companies that develop cutting-edge technologies and automation platforms adapted their technologies for COVID-19 screening. As an example, Hamilton offered an automated RNA extraction solution with its MagEx STARlet platform and also an automated qPCR mix prep solution with PCR Prep STARlet (Hamilton Company, USA). Besides these, the Fluent and Freedom EVO platforms of Tecan were adapted for use to automate RNA extraction and PCR preparation processes (Tecan, Switzerland). Liquid handling platforms such as the CyBio FeliX (Analytik Jena, Germany) and Echo 525 (Labcyte Inc., USA) were shown to be easily integrated into a SARS-CoV-2 automated workflow, nevertheless the accessibility of all the aforementioned equipment was limited to a few laboratories because of high pricing.

Some other companies like Hologic (USA) and Roche (Switzerland) offered equally expensive options with the added disadvantage that they predominantly use proprietary and expensive reagents/ reagent cartridges, causing a decrease in access and flexibility between different protocols. Due to this, the community-driven “Free and Open-Source Scientific and Medical Hardware” (FOSH) rose to action to offer cheaper, reliable, and customisable platforms. FOSH follows the same rules as open source software which consist in offering “blueprints” for a specific tool in a manner where every user can study, learn, share, customise and even commercialise a specific tool or protocol for any particular application (32). For laboratory automation a recent popular example is the Opentrons' OT-2 platform. This platform offers an affordable and open-source lab automation system that allows complete user customisation, including the potential for scaled-up molecular diagnosis reactions. Opentrons developed its own population-scale SARS-CoV-2 testing procedure involving three steps operated by OT-2 robots and one RT-qPCR step (Opentrons, USA). In this workflow, sample plating was the first step performed, using an OT-2 to transfer samples from the collection tubes into a 96 well-plate. The second step applied an RNA extraction process by using a magnetic module. Finally, the RNA isolated from the samples were prepared for an RT-qPCR task, to be completed separately in a different room to avoid cross contamination. To scale up this workflow to 2,400 samples per day a set-up with ten OT-2 robots working simultaneously was proposed by the company (Opentrons, USA).

A thriving GitHub community (https://github.com/Opentrons, 01/24/2022) is available to develop and share custom scripts for precise operation of the robot. At the same time, several SARS-CoV-2 related protocols including RNA extraction are available at protocol.io (https://www.protocols.io/groups/opentrons-covid19-testing, 01/24/2022). One of the key advantages of using the OT-2 platform is its affordable price compared to other high-throughput automation platforms. It is also highly customisable and easy to operate. However, despite the many positives, OT-2 platforms have some important limitations to consider, including: the lack of control mechanisms to detect clot formation or sample volume, lack of many specific modules such as de-lidders, incubators, centrifuges, tube capping/de-capping and a lack of hardware to ensure sample tracking which is essential for quality standards (33). Nevertheless, many of these functionalities can be added using opensource external instrumentation, but advanced programming skills are required for to successfully implement these tools (34).

**Figure 4:**
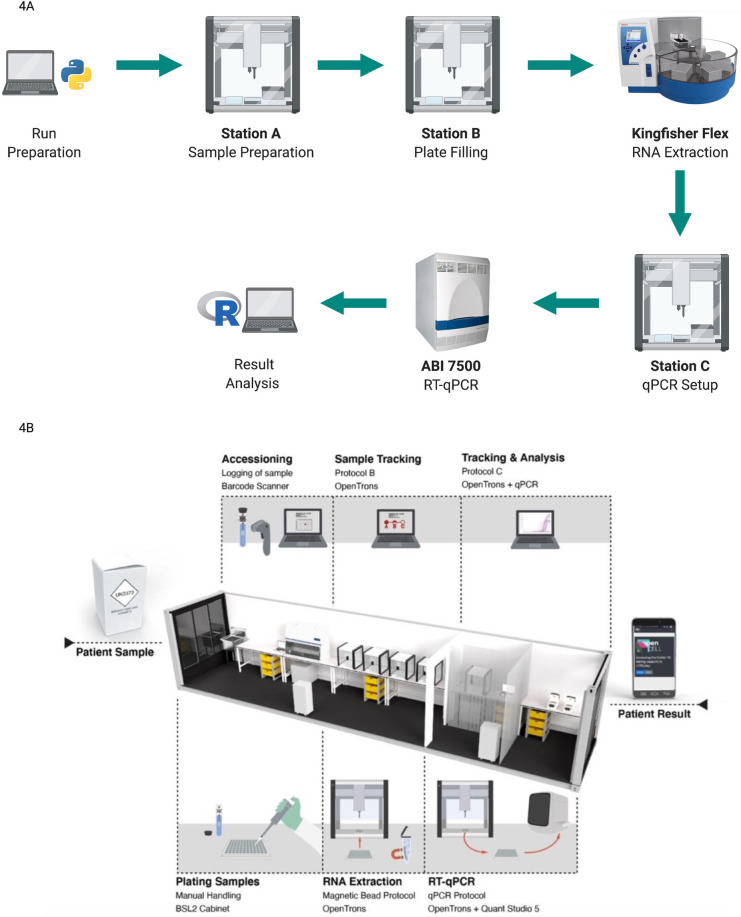
(created with BioRender.com). Different protocols using the open-source OT-2 automated platform for COVID-19 testing (**A**) automated workflow Designed at CBD. An initial run preparation was performed using open-source Python coding. Initial sample setup, sample preparation, and plate filling and qPCR mix preparation were performed by OT-2 robots. RNA extraction was processed by KingFisher Flex and RT- qPCR was run by ABI 7500. Analysis results were exported as a user-friendly R file. Figure adapted from Villanueva-Cañas et al. (33) (**B**) the layout and workflow of a semi-automated CONTAIN lab. Three separate sections were used for plating samples, RNA extraction and RT-qPCR respectively. OT-2 robots were used for the RNA extraction process and qPCR mix preparation. Station C which is used for the RT-qPCR run also contained two subsections to separate OT-2 and qPCR devices. Image taken from OpenCell.bio, Walker et al. (35).

The Biomedical Diagnostic Centre (CBD) of the Hospital Clinic of Barcelona has recently obtained the green light from the FDA to use Opentrons for fast, high-throughput SARS-CoV-2 screening with a capacity of 2,400 test per day (testing speed is 96 samples in 4 h with a re-start time for a new cycle every 70 min) (Opentrons, USA). The workflow designed by researchers at CBD utilised four Opentrons OT-2s, one KingFisher Flex extraction instrument by ThermoFisher and one ABI 7,500 qPCR device (33) as shown in process diagram in Figure 4A. The procedure included the initial setup, sample preparation, plate filling for RNA extraction and qPCR mix preparation. RNA extraction and real-time qPCR were carried out by KingFisher Flex (Thermo Fisher Scientific, USA) and ABI 7,500 qPCR (Applied Biosystems, USA) respectively while all the other tasks were performed by the OT-2 robots. The European Molecular Genetics Quality Network provided an external quality assessment comparing this system and the Roche, Cobas 6800, and Hamilton-Seegene platforms. The results of the external assessment showed consistent Ct levels (which are inversely proportional to the amount of target nucleic acid in the samples) between the system developed and other similar platforms (33).

## Mobile and high-throughput testing facilities

The strategy of track and trace has been a very significant part of the SARS-CoV-2 fight, (35), leading researchers to develop innovative solutions to increase the access of automated molecular screening workflows. As an example, a modular and mobile Biosafety Level 2+ laboratory called CONTAIN was developed for automated molecular testing of SARS-CoV-2, taking advantage of the versatile OT-2 platforms (35). This mobile lab was set up in 40 ft shipping containers, which each held five OT-2 robots and performed RT-qPCR-based diagnostics with a maximum daily testing capacity of 2,400 tests. The CONTAIN lab consisted of three separated stations; Station A for unpackaging and logging of samples, Station B for an RNA extraction step completed by four OT-2 robots, and finally Station C containing one OT-2 and two qPCR devices for the RT-qPCR run. Figure 4B represents the general layout and workflow of a CONTAIN lab. For the RNA extraction process, the open-source Bio-On-Magnetic-Bead (BOMB) protocol, which utilises magnetic beads, was adapted to run in an OT-2. In the CONTAIN lab workflow the initial step of sample plating was done manually, allowing processing of the same number of samples as the solution provided by Opentrons (2,400 per day) in a semi-automated way using five OT-2s instead of the 10 recommended by Opentrons. Compared with the clinical results, CONTAIN values showed a strong correlation with *R*^2 ^= 0.7698 on 30 patient samples, highlighting the effectiveness of mobile automation (35).

This innovative approach of a mobile/container laboratory benefiting from open-source automation platform highlights some important potential: a mobile lab could be shipped between cities or even around the world—allowing rapid deployment in virus hotspots globally. In addition, the containers could be stacked to build larger facilities depending on testing requirement (35).

## Low-cost bio-automation SARS-CoV-2 testing constraints

### Cost of the equipment

When it comes to automation, the cost of the platforms is usually the first hurdle to overcome (36), particularly for low-resource settings. Prices of equipment may vary widely between platforms starting from a few thousand dollars in the cheapest cases up to nearly a quarter million dollars for the most advanced alternatives (Synthace, UK).

As mentioned in previous sections, the OT-2 robot by Opentrons is one of the most affordable platforms, however, the initial price is still nearly $6,000 USD (as of 2020) with the most basic option only including one single channel pipette and one multichannel pipette. Additionally, while the price of the robot alone can be a significant limitation, the basic configuration is not sufficient to completely automate COVID-19 testing. Extra proprietary modules which are sold separately (i.e., thermal cycler, temperature, and magnetic modules, etc.) are necessary for automated COVID-19 testing and increase the overall cost to $15,000 USD, making even this low-cost platform an unaffordable option for low-resource laboratories.

### Cost and access of consumables

Consumables and access to them are another important constraint to consider before implementing an automation testing platform. One key issue is that most automation platforms require the user to use their own consumables (34). Pipette tips, tube racks, containers, and reagents are in many cases—custom made for each particular equipment and must be bought directly from the manufacturer at inflated prices, imposing additional costs (taxes, shipping, etc.) to acquiring these supplies (34, 36). In addition to the cost, another critical concern associated with consumables is the limited access to these supplies caused by global shortage or distribution issues. In some cases, due to the sudden increase in demand, some consumables (proprietary pipette tips, racks, containers or tubes) are commonly out of stock for weeks and the waiting time for receiving them can be extremely long (36).

Reagent supply is a significant barrier to adopting automated testing for many laboratories— even those who possess an OT-2 or other affordable platforms. This also applies to detection of SARS-CoV-2 nucleic acids as most protocols require highly specialized and expensive reagents. Excluding the economic side, the limited access to specialised reagents and the frequent requirement for cold transport and storage (37) impose additional limitations on performing high-throughput nucleic acid testing, particularly in low-resource settings.

In order to reduce the dependency on expensive and proprietary consumables— and also as an alternative to relieve supply dependency from the manufacturer— efforts are being made towards utilising 3D printing to develop affordable and compatible alternatives. In general, the major equipment (robot) is designed to use proprietary consumables, nevertheless, some low-cost automation machines are also compatible (or can be adapted with minor modifications) with consumables that are regularly used in many biomedical laboratories. This is particularly true for plastic tips, racks and 3D printed containers (38). Additionally, there is already an Opentrons github community (https://github.com/Opentrons, February 2021) that has come up with 3D printing design ideas for different necessities. These communities usually offer their designs for free so that the user can try them and make modifications as they see fit.

### Software for automation, data analysis and user interface (UI)

Another important factor for adopting an automation testing platform is the general requirement of at least basic programming skills to operate low cost robots or intermediate programming skills to perform more complex tasks (34). The majority of “wet lab” researchers use computers as data analysis tools that are often performed in specialised software and do not require any programming skills. While the established automation platforms mentioned before include a robust and easy to use user interface to perform basic and advanced operations, most of the low-cost automation platforms generally only include a relatively simple user interface for designing and performing basic operations. Generally, an application program interface (API) is included in Python allowing the user to code detailed instructions to the liquid-handling robot to perform more complex tasks. It is important to remark that therefore, for advanced customisation and flexibility in the low-cost automation platforms, programming skills are absolutely necessary to take advantage of the full potential of automation and data analysis. Researchers interested in automation will indeed need to have an “amphibious” set of skills consisting of both “wet” and “dry” biology and programming skills respectively to effectively work with automation (39).

### Nasopharyngeal swabs sampling bottleneck

Ramping up testing for effective SARS-CoV-2 surveillance has faced several barriers. One of these is the reliance on nasopharyngeal swabs (NP), as sampling with swabs can be uncomfortable for people, discouraging them from getting tested frequently. The post-processing of NP is also difficult to automate (40). NP swabs also have to be collected by a trained individual, adding a logistical barrier and putting countries with less logistic support at high risk of getting their testing staff infected (40, 41). In addition, when SARS-CoV-2 spread worldwide at rapid pace, it was reported that countries suffered intense strain on the healthcare consumable supply chain (i.e., Swabs) (42, 43). An alternative proposal to alleviate the scarcity of swabs is the application of 3D printing technology to produce them (43, 44).

Saliva sampling has emerged as a more suitable option for low-resource and remote settings. Saliva sampling is a simple approach with the potential to drive down costs, while also relieving pressure from the consumable supply chain and promises to facilitate more effective testing due to the safe and non-invasive nature of its collection. It is also highly compatible with an automated approach and finally saliva samples contain high viral load (45). Recognising these benefits, the FDA approved a saliva collection and preservation device for downstream COVID-19 testing (45). Direct comparison of saliva to nasopharyngeal (NP) swabs from the same individuals revealed that saliva samples could provide similarly consistent and sensitive results for COVID-19 detection (YALE, School of Public Health, https://publichealth.yale.edu/salivadirect/, April 2021).

In order to quickly inactivate/lyse virions while also protecting their RNA from endogenous RNAses in the saliva sample, a simple protocol was implemented utilising a shelf-stable reducing agent, tris(2-carboxyethyl) phosphine (TCEP), combined with the divalent cation chelator ethylenediaminetetraacetic acid (EDTA) and a brief period of heat (95 °C) (46). This protocol is called HUDSON (heating unextracted diagnostic samples to obliterate nucleases) and is compatible with most technologies described in Figure 1 as process of extraction and amplification of RNA. Saliva was collected in a viral transport media tube and transported to the laboratory for analysis where a simple RNA extraction was performed which did not require expensive RNA extraction kits. A solution of TCEP (100 mM) and EDTA (1 mM) was added to the sample. A mild heat treatment consisting of 50°C for 5 min and 64° for 5 min was employed. Diluted TCEP/EDTA is compatible with LAMP (45) hence it could also be employed with RT-RPA.

It is important to mention, that from personal preliminary work, it has been found that the re-detection rate based on NP can be lower than that by saliva. This can be explained by the fact that q-PCR made from saliva samples often lead to inaccurate results consequence of a failure during saliva sampling which derive in a need for double checking for re-detection. In addition to this, different saliva samples can have different viscosity. In order to ensure consistent, automated pipetting of saliva, it may be necessary to dilute those samples, thus influencing the test and decreasing the speed of the aliquoting step and sensitivity of the analysis. Therefore, saliva viscosity for consistent automated diagnostic precision requires further investigation.

## The role of high-throughput testing in the detection of new variants

Since the beginning of the pandemic the diagnostics strategies used by different governments have varied across different times. According to Mercer et al. (47), there are normally 5 phases of testing during the pandemic: zoonotic transmission, global spread, outbreak, community transmission and regional/seasonal outbreak. During the last 3 phases of the COVID19 pandemic, population-scale testing was carried out by RT-qPCR using automated facilities and equipment like the ones described in the previous sections, with the addition that routinely a proportion of positives samples were subjected to whole-genome sequencing for surveillance of existing and new variants (47).

The Rosalind Franklin Laboratory in Royal Leamington Spa is an example of a massive testing facility used for detection of new variants. This facility has a processing capacity of 400,000 PCR tests a week and it couples it with genomic sequencing capabilities for the detection of new variants of concern (VOC) (48). They make use of an ultra-high throughput PCR Nexar workflow which enables up to 150,000 tests per day per system, making it the highest PCR testing capacity per system worldwide (49).

Another important example is found in the UK Lighthouse Labs Network at Alderley Park, were 8 million samples were analysed by RT-qPCR in only 10 months with a capacity of 80,000 samples per day (11). For this testing strategy, 3 viral regions of SARS-CoV-2 virus were targeted: N, S and Orf1ab (11). To allow massive testing, nucleic acid extraction was performed by a Kingfisher Flex extraction platform (Thermo Fisher Scientific, USA) while amplification was carried on a Quantstudio Flex 7 System (Thermo Fisher Scientific, USA). Positive results were tracked using the postal district of sample origin which allowed to track how the mutation was spreading through the UK. When testing started in April 2020 the 3 regions examined by PCR would show a positive result. The consistency in the results within the same sample indicated that the same viral strain was present among all samples tested. However, by September of the same year there was an increase in samples testing negative for the S region but still positive for N and Orf1ab regions. The change from positive to negative in one of the regions of the virus with respect to the initial samples suggested a mutation of the virus. Whole-generation sequencing confirmed a new lineage (B.1.1.7 better known as Alpha) variant which was designated as a Variant of Concern (VOC). As larger scale automated testing continued, by January of 2021, 70% of daily samples corresponded to this variant while the number increased to 98% by February (11).

Finally, it is very relevant to mention that apart from the UK Lighthouse Labs Network –dedicated to COVID-19 testing for NHS test and Trace—the Coronavirus Disease 2019 (COVID-19) Genomics UK Consortium (COG-UK) has enable an important genomic epidemiology database by developing high-throughput sequencing and analysis workflows of thousands of SARS-CoV-2 genomic sequences (50). COG-UK has created a web resource that allows the analysis of viral mutations and variants in the UK. The repository contains millions of sequences that enable in-silico surveillance of new variants (51).

### Integration of NSG into automated COVID-19 diagnostics

As sequencing technologies improve and reduce in cost, they have become stronger candidates as an alternative for mass clinical testing. Next Generation Sequencing (NGS) is one of these technologies with a variety of potential applications, including metagenomic NGS (mNGS), allowing for an unbiased approach to the detection of pathogens. A great advantage of mNGS is unbiased sampling which enables every species in the sample, leading to identification of unexpected and even unknown pathogens (52). This technology was crucial for identification and characterization of SARS-CoV-2 genome (47, 50). Automation has also been used to further increase the capacity of detection of such NGS workflows where different automation devices have been used in tandem to diagnose clinical microbiological samples.

A recent strategy published by researchers from the United States gives a perfect example of how NGS can be coupled with a high throughput workflow for the identification potential diagnostic and therapeutic genes for SARS-CoV-2 (50). The workflow consists in 4 steps described as follows.

1. Extraction of viral RNA, cDNA preparation and amplification by PCR. All of these steps made use of Agilent's Bravo robot (USA)
2. PCR products were purified using BlueCatBio's (Germany) Bluewasher and pooled into a single library using Hamilton's (USA) Startlet. Amplicons were separated by size using Sage Science's (USA) Blue Pippin.
3. Library was sequenced using Illumina's NovaSeq 6000.
4. Bioinformatic analysis using an in-house developed bioinformatic pipeline.

It is important to mention that this is not the first method reported for the purpose of identifying genes of interest for SARS-CoV-2 but it is the first that overcomes the limitations of the workflow—that often limits the scalability of the process— by the integration of various liquid handling robots (50).

## Conclusions

Automation is now an inseparable part of modern society as it eases many processes that are encountered during everyday life. The studies mentioned in this report have highlighted the impact that automation of molecular diagnosis processes have had on increasing the capacity for COVID-19 testing using RT-qPCR. Alternative methods which would require less complex equipment based on CRISPR technologies have shown to be easily automated, although further validation is needed before being fully implemented for mass testing. Thus, it is apparent that partnerships must be built between companies and academia working in a variety of fields to develop more powerful solutions for automated diagnosis workflows for infectious diseases.

Some of the most important challenges to be addressed in this field include the capital cost of equipment and the cost and accessibility for healthcare consumables. Open-source approaches are democratising access to automation, however there are still many bottlenecks to overcome, including the advanced computing skills required to operate such automation platforms. Nasopharyngeal swabbing was another obstacle to full automation as at present this process requires significant human involvement. Saliva sampling seems to a solution to this bottleneck as it requires less consumables and it is more practical to automate, however, more studies are needed to validate automated saliva sampling, specially using low-cost automation.

From all the protocols and equipment discussed, the selection of one of them may vary depending on the capabilities and requirements of each laboratory. Samples size, budget, expertise of the personnel and availability of equipment are factors that should be considered when choosing the correct workflow for high-throughput diagnostic. RT- PCR and RT- LAMP remain as the golden standard due to their accuracy, availability of reagents and well proven efficiency. Nevertheless, CRISPR-based technologies have a huge potential for lower cost automation and their versatility provide a huge potential to be used in marginalised to be used as a point-of-care testing technology.

The use of automation is definitely becoming the norm for high-throughput diagnostics, where low cost open-source automation has reached the right level of maturity to accelerate and democratise the access of such tools to a wider audience.
